# Cost-effectiveness of transcatheter aortic valve implantation in patients at low surgical risk in France: a model-based analysis of the Evolut LR trial

**DOI:** 10.1007/s10198-023-01590-x

**Published:** 2023-05-30

**Authors:** Didier Tchétché, Coline Dubois de Gennes, Quentin Cormerais, Benjamin P. Geisler, Camille Dutot, Fanny Wilquin-Bequet, Manon Breau-Brunel, Béranger Lueza, Jan B. Pietzsch

**Affiliations:** 1 Clinique Pasteur, 45 Avenue de Lombez, 31300 Toulouse, France; 2 Amaris, London, UK; 3 Medtronic, Plc, Dublin, Ireland; 4 Massachusetts General Hospital, Harvard Medical School, Boston, MA USA; 5 Wing Tech Inc., Menlo Park, CA USA

**Keywords:** Aortic valve stenosis, Transcatheter aortic valve implantation, Surgical aortic valve replacement, Cost–benefit analysis, Health-related quality of life, France, I130, I180, H51

## Abstract

**Background:**

In the recent Evolut Low Risk randomized trial, transcatheter aortic valve implantation (TAVI) was shown to be non-inferior to surgery (SAVR) regarding the composite end point of all-cause mortality or disabling stroke at 24 months.

**Aims:**

To evaluate the cost-effectiveness of self-expandable TAVI in low-risk patients, using the French healthcare system as the basis for analysis.

**Methods:**

Mortality, health-related quality of life, and clinical event rates through two-year follow-up were derived from trial data (*N* = 725 TAVI and *N* = 678 SAVR; mean age: 73.9 years; mean STS-PROM: 1.9%). Cost inputs were based on real-world data for TAVI and SAVR procedures in the French healthcare system. Costs and effectiveness as quality-adjusted life years (QALYs) were projected to lifetime via a decision-analytic model under assumption of no mortality difference beyond two years. The discounted incremental cost-effectiveness ratio (ICER) was evaluated against a willingness-to-pay threshold of €50,000 per QALY gained. Deterministic and probabilistic sensitivity analyses were conducted, including assumptions about differential long-term survival.

**Results:**

For the base case, mean survival was 13.69 vs 13.56 (+ 0.13) years for TAVI and SAVR, respectively. Discounted QALYs were 9.34 vs. 9.21 (+ 0.13) and discounted lifetime costs €52,267 vs. €51,433 (+ €833), resulting in a lifetime ICER of €6368 per QALY gained. In probabilistic sensitivity analysis, TAVI was found dominant or cost-effective in 74.4% of samples.

**Conclusion:**

TAVI in patients at low surgical risk is a cost-effective alternative to SAVR in the French healthcare system. Longer follow-up data will help increase the accuracy of lifetime survival projections.

**Graphical Abstract:**

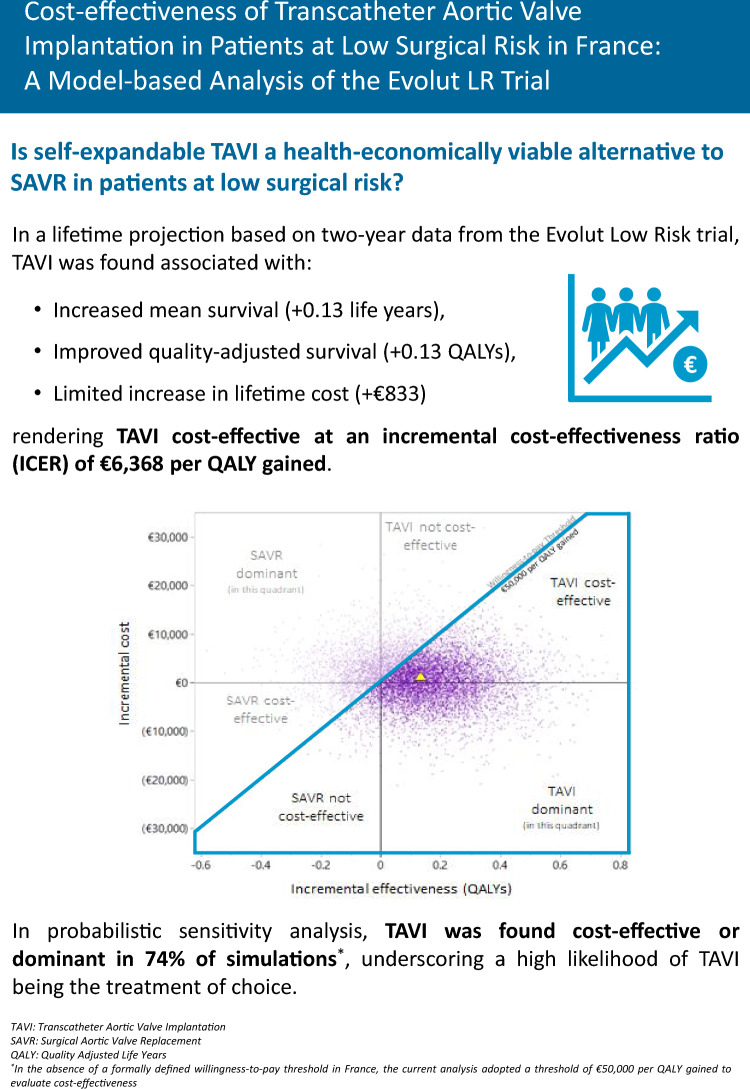

**Supplementary Information:**

The online version contains supplementary material available at 10.1007/s10198-023-01590-x.

## Introduction

Over the last decade, the clinical indication for transcatheter aortic valve implantation (TAVI) in patients with severe aortic stenosis has seen continuous expansion from patients at extreme and high risk of perioperative death to patients at intermediate risk and, most recently, those at low surgical risk. Two multi-center trials have provided data about the safety and effectiveness of TAVI as an alternative to surgical aortic valve replacement (SAVR) in patients at low surgical risk: PARTNER 3 and Evolut Low Risk (LR), evaluating a balloon-expandable (SAPIEN, Edwards Lifesciences, Irvine, CA, USA) and a self-expandable valve (Evolut R and PRO, Medtronic Inc., Mounds View, MN, USA), respectively [1, 2]. Based on these findings, the latest iteration of the European ESC/EACTS guidelines recommend TAVI as the preferred treatment in low-risk patients age 75 or higher who are deemed suitable for transfemoral TAVI placement upon heart team assessment, as well as those unsuitable or at high risk for SAVR (Level I-A recommendation) [3].

While the body of clinical evidence about TAVI in low-risk populations is reasonably comprehensive—albeit still emerging with longer follow-up data—less is known about the expected cost-effectiveness of TAVI in this new indication. The current study assessed the cost-effectiveness of self-expandable TAVI versus SAVR in a European context based on latest data from the Evolut LR trial, using the French healthcare system as the analysis setting.

## Methods

### Overview

A decision-analytic Markov model was developed to project strategy-specific outcomes and costs over the patients’ lifetime. For the first two years, events and survival were modeled ‘along-the-trial’ based on trial-reported evidence, followed by a long-term projection that assumed no difference in survival and health-related quality of life between the TAVI and SAVR cohorts beyond two years in the base case scenario. The general structure of the analytical model followed those used in two previously published decision-analytic models of the CoreValve High Risk trial and of the Nordic Aortic Valve Intervention (NOTION) trial [4, 5]. Costs, including for the index treatment and adverse event management, were based on real-world data and national tariffs from the French healthcare system, literature and experts’ opinion, and were calculated in a collective perspective following guidance from the French National Authority for Health (Haute Autorité de Santé – HAS) [6].

### Model structure

The Markov model included three primary health states—alive with no stroke, alive post-stroke, and death—a transitory state for acute stroke, and several nested substates that captured individual adverse events (AEs) that might occur in each cycle. These included myocardial infarction (MI), permanent pacemaker implantation (PPI), bleeding event (major or life-threatening/disabling), cardiogenic shock, acute kidney injury (AKI), major vascular complication, atrial fibrillation (AF), aortic valve hospitalization, and reintervention. Stratification by stroke status was applied as stroke events have the most pronounced effect on patient quality-of-life and long-term post-event cost [7]. The quality-of-life and cost implications of all other events were embedded in the no-stroke and post-stroke states. The model had a cycle length of one month, with the first cycle capturing pre-operative work-up, index procedure and hospitalization, as well as rehabilitation where applicable. Through 24 months, monthly event rates were derived from trial data [2]. Beyond two years, mortality and stroke events were the only adverse events considered to capture long-term efficacy in model projections in the base case analysis, with post-event costs of stroke and PPI applied annually.

### Clinical events and health-related quality of life

Clinical event probabilities and health-related quality of life (HRQoL) measurements were derived from the Evolut Low Risk Trial [2, 8]. In brief, this multinational, randomized, noninferiority clinical trial compared the safety and efficacy of TAVI with a self-expanding bioprosthesis (Evolut R and PRO, Medtronic Inc., Mounds View, MN, USA) to SAVR (size and type of surgical valve at surgeons’ discretion, candidates for mechanical valves excluded) in patients at low surgical risk of death [2]. The study enrolled a total of 1,468 patients, and *N* = 725 and *N* = 678 participants were ultimately treated with TAVI and SAVR, respectively. The mean age of participants was 73.9 years, and 35.3% were female. The Society of Thoracic Surgeons Predicted Risk of Mortality (STS-PROM), which provides an estimate of the risk of death at 30 days among patients undergoing SAVR based on several demographic and procedural variables, was 1.9 ± 0.7% [2]. The available patient-level data included follow-up at 30 days, six, twelve, and 24 months after index treatment. Table 1 shows the key input parameters, with complete information provided in the supplementary materials.

**Table 1:** Input parameters. Cohort characteristics, clinical event rates, costs, and utility for TAVI and SAVR used in analysis model

	30-day		12 months		24 months			
Clinical event probabilities (cumulative)	TAVI	SAVR	TAVI	SAVR	TAVI	SAVR	Acute treatment cost (events beyond first 30-days**)	Considered follow on cost (annual)
Death	0.4%	1.2%	2.1%	2.7%	3.5%	4.4%		
Stroke	3.4%	3.2%	4.3%	4.3%	6.2%	6.0%	€5857	€8530
Myocardial infarction	0.8%	1.3%	1.8%	1.6%	2.1%	1.6%	€5776	
Permanent pacemaker implantation	17.1%	6.0%	19.0%	6.8%	21.8%	7.7%	€6734	€545 (yr. 1)
								€273 (yr. 2 +)
Bleeding event	6.6%	10.4%	8.1%	12.2%	10.4%	13.7%	€11,282	
Cardiogenic shock	0.0%	0.0%	0.0%	0.0%	0.0%	0.0%	€4789	
Acute kidney injury	2.1%	10.1%	2.1%	10.1%	2.1%	10.1%	€4589	
Major vascular complication	3.7%	3.1%	3.7%	3.4%	3.9%	3.6%	€7511	
Atrial fibrillation	7.5%	35.5%	9.4%	38.7%	12.2%	39.7%	€3608	
Aortic valve rehospitalization	1.2%	2.4%	3.3%	5.9%	4.9%	7.2%	€4789	
Reintervention (surgical)	0.3%	0.3%	0.6%	0.4%	0.9%	0.9%	€23,324	

Index procedure costs	TAVI	SAVR						
Pre-operative care	€3141	€3112						
Procedure and index hospitalization	€23,743	€23,324						
Rehabilitation (based on utilization)	€74	€652						

Outpatient follow-up cost	Month-1		Month 6		Month 12		Annually beyond 12 months	
	TAVI	SAVR	TAVI	SAVR	TAVI	SAVR	TAVI	SAVR
	€237		€60﻿		€18		€262	

Utilities per post-procedure period	Month 1		Month 6		Month 12		Month 13 +	
	TAVI	SAVR	TAVI	SAVR	TAVI	SAVR	TAVI	SAVR
Alive, no stroke	0.847	0.768	0.848	0.826	0.825	0.839	0.832	
Alive, post-stroke*	0.805		0.789		0.792		0.792	

See supplementary materials for detailed sources, parameter ranges, and distributional information

TAVI, transcatheter aortic valve implantation; SAVR, surgical aortic valve replacement

*No distinction in utilities in patients with strokes after TAVI or SAVR because of small number of patients in these subgroups

**Except for aortic valve rehospitalization occurring during first 30 days and pacemaker implantations performed in first 30 days after patient has been discharged from index hospitalization, all first month costs are considered to be included in the index cost

HRQoL estimates were based on trial-collected EuroQoL-5D-5L (EQ-5D) values measured at baseline, 1 month, 6 months, and one year. These index values were converted to utility estimates using a France-specific scoring algorithm developed for the EQ-5D with five levels [9]. Strategy-specific utilities for the “alive with no stroke” states were derived for the TAVI and SAVR arms of the model. As the twelve-month utility estimate did not differ between the strategies, the same utility of 0.832 was assumed to be maintained for both strategies beyond one year. EQ-5D data informing the “alive post-stroke” states were limited based on the small proportion of patients suffering a stroke during the one-year follow-up period in which EQ-5D data were collected. To obtain a reasonably robust estimate, the utility for this health state was calculated based on post-stroke data from both strategies. As in the no-stroke state, the twelve-month utility estimate was maintained for all future periods in the model projection. All clinical input parameters and HRQoL were analyzed in SAS 9.4 (SAS Institute, Cary, NC, USA).

### Resource use and costs

The input parameters for costs are provided in Table 1, with further detail provided in Table S.2.1 in the supplementary materials. As defined by French methodological guidelines for cost effectiveness analysis, the economic analysis was conducted from a perspective which includes direct costs incurred by all relevant stakeholders, referred to as the “collective” perspective [6]. Costs considered in the analysis included preoperative cost, index procedure and hospitalization cost, rehabilitation cost, follow-up costs covering necessary visits and exams for all patients, acute costs of adverse event treatment and adverse event follow-up costs, transportation cost, and end-of-life cost. Index procedure and hospitalization cost including device and materials were derived from the latest available data year of the French National Cost Study (ENC, Etude Nationale des Coûts) [10] and from public data including tariffs. The TAVI strategy index cost, for analysis purposes, considered a TAVI device cost of €15,419, per published tariff and as applicable in 2019. Cost of adverse event treatment within the first 30 days was assumed to be covered with the index hospitalization cost, except for aortic valve hospitalizations and pacemaker implantations conducted outside the index stay. Cost for acute treatment of adverse events beyond the first month was again based on the French National Cost study [10]. Long-term follow-up costs related to AEs were considered for stroke and pacemaker events [11, 12]. Where needed, costs were inflated to 2019—the data year for this analysis—using the consumer price index [13]. In the analysis, costs and outcomes were discounted at 2.5% per annum, per current methodological guidelines for France [6].

### Model outcomes and willingness-to-pay threshold

The primary analysis outcomes were strategy-specific treatment costs, QALY gains, and the resulting incremental cost effectiveness ratio (ICER), measured in euros per QALY gained. Further, stratification of total cost by cost type (preoperative work-up, index treatment incl. AE and rehabilitation, subsequent adverse event and follow-up, other follow-up and management cost) was provided.

In the absence of a formally defined willingness-to-pay threshold in France, the current analysis adopted a threshold of €50,000 per QALY gained to evaluate cost-effectiveness. This value is in line with the World Health Organization’s recommendation to consider one to three times the country-specific per-capita gross domestic product, which in France was most recently reported as €30,610 in 2020 [14, 15].

### Scenario, sensitivity and threshold analyses

The effects of parameter uncertainty on the cost-effectiveness results were evaluated in deterministic sensitivity analyses by varying each parameter across ranges derived from the distributional information available for each clinical and cost parameter. For cost parameters for which no distributional information was available, a range of ± 10% of the base value was explored (see supplementary materials). The effect of using an alternative France-specific three-level scoring algorithm for EQ-5D utilities was also evaluated [16]. Several scenarios were explored, including consideration of all adverse events beyond two years at the rate observed between 12 and 24 months, analysis horizons shorter than lifetime, evaluations of the effect of different survival between TAVI and SAVR based on relative mortality risks observed between six and 24 months and twelve to 24 months and two hypothetical scenarios. Further, to complement the base case scenario that assumed the same quality of life for TAVI and SAVR in the no-stroke state based on no statistically significant difference in EQ-5D observed at twelve months, two scenarios explored numerical differences in quality of life were maintained over the remaining lifetime.

A probabilistic sensitivity analysis (PSA) was performed to study the overall effect of parameter uncertainty on the results. For the PSA, all parameters were varied simultaneously in a total of 10,000 simulation runs, with parameter values drawn in each cycle from the defined probability distributions. To evaluate results, a cost-effectiveness scatter plot and the resulting cost-effectiveness acceptability curve were generated.

### Model validation

Internal validity of the analysis model was evaluated through review and detailed checks by a second group of health economists not involved in the programming of the original model. External validity, primarily related to survival assumptions and projections, was conducted comparing the projections to data from prior studies with longer follow-up and to country-specific population survival.

## Results

### Survival

**Fig. 1:**
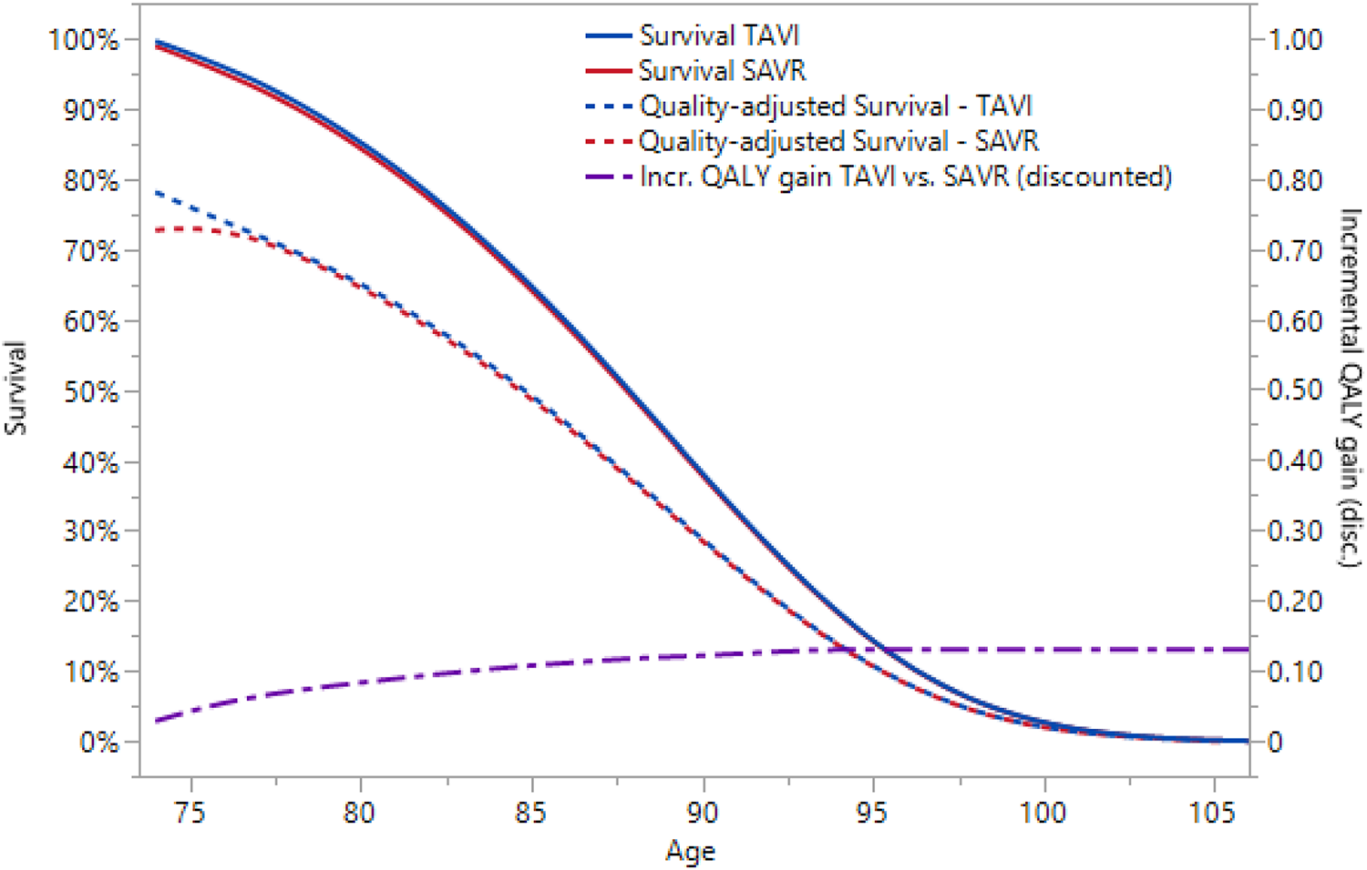
Survival projections and incremental QALY gain. Curves show the survival and quality-adjusted survival curves projected in the base case analysis, and the resulting discounted cumulative incremental QALY gain

Model-projected undiscounted survival for TAVI and SAVR was 13.69 and 13.56 (+ 0.13) life years (LYs), respectively. Undiscounted quality-adjusted survival was 11.39 and 11.24 (+ 0.15) QALYs, and discounted quality-adjusted survival was 9.34 and 9.21 (+ 0.13) QALYs. The cumulative QALY gain in the first year was primarily driven by differences in HRQoL observed between the TAVI and SAVR strategies (Fig. 1).

### Costs

**Fig. 2:**
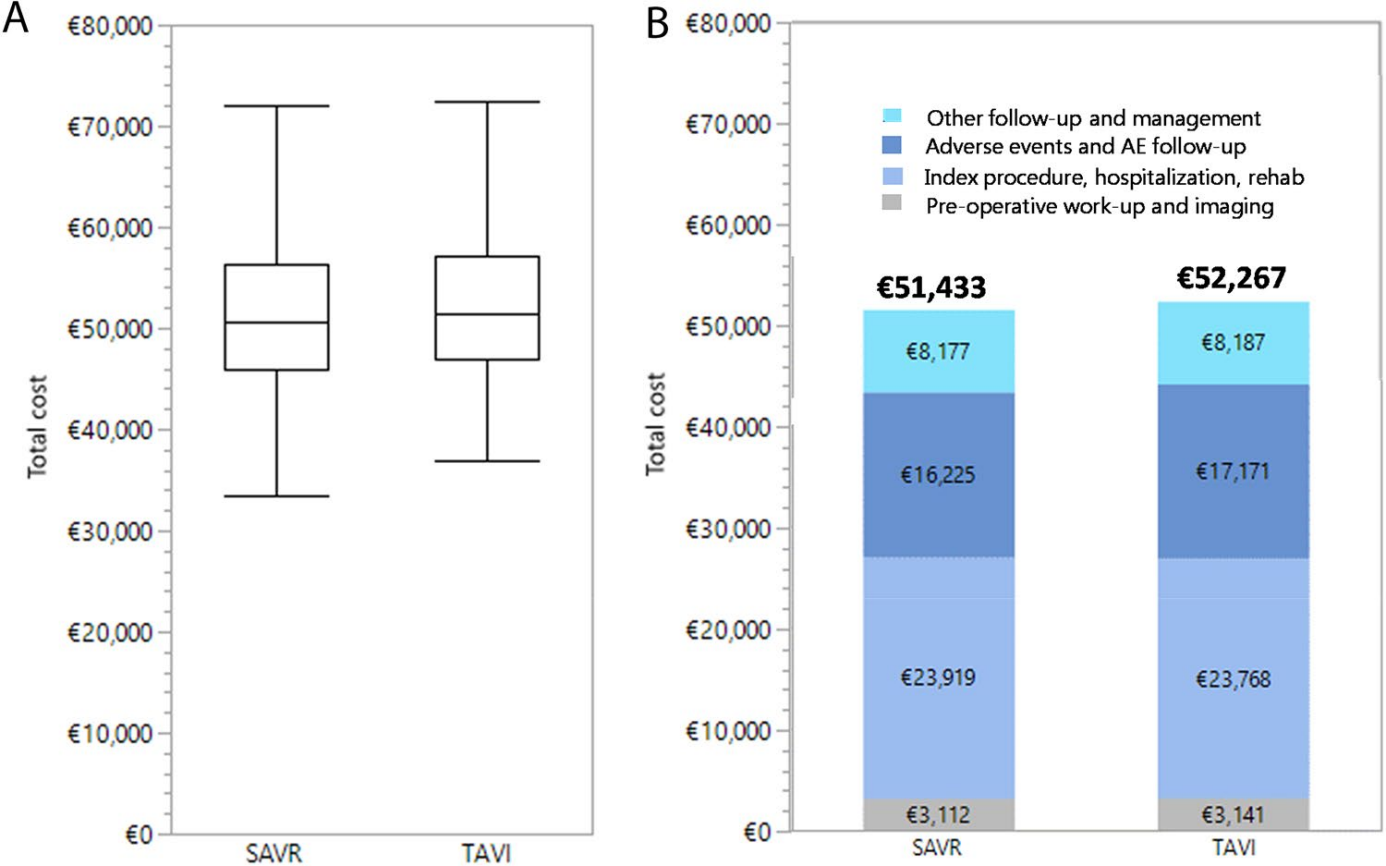
Lifetime Costs of SAVR and TAVI. Boxplot of lifetime costs of SAVR and TAVI strategy derived from probabilistic sensitivity analysis (**A**) and breakdown of deterministic base case lifetime cost by cost type (**B**)

Cumulative discounted lifetime costs were €52,267 and €51,433 (+ €833) for TAVI and SAVR respectively. Model projected mean preoperative costs were €29 higher, index treatment cost including adverse events and rehabilitation €151 lower, subsequent AE and AE follow-up cost €946 higher, and other follow-up and maintenance cost €10 higher in the TAVI as compared to the SAVR strategy. The 95% confidence intervals of the lifetime costs, derived from the 10,000 PSA simulation runs of the analysis model, were (€41,114–€72,562) for TAVI and (€39,651–€71,839) for SAVR (Fig. 2).

### Base case, sensitivity, and scenario analyses

The projected higher lifetime cost of TAVI, in conjunction with its projected incremental gain in QALYs, resulted in a lifetime ICER of €6368 per QALY in the base case, and thus cost-effective. The corresponding ICER measured in € per LY gained was only slightly higher at €7828. While shorter analysis horizons led to lower incremental QALY gains, incremental costs were also lower, resulting in a relatively stable ICER across various tested follow-up horizons. In an extreme scenario of a 2-year analysis horizon—that was only explored to assess the QALY and cost difference at the end of the current available trial follow-up, yielded a theoretical ICER of €2262 per QALY gained, approximately one-third of the base case ICER. Consideration of all other AEs beyond stroke and death not only in the first two years, but also beyond, increased the ICER minimally. Scoring of the trial-collected EQ-5D utilities with the alternative French scoring algorithm for EQ-5D-3L [16] led to somewhat lower lifetime QALYs in both strategies, but minimally higher incremental QALY gain of 0.14 over lifetime, resulting in a minimally lower ICER than the base case. Maintaining the—not statistically significant— numerical difference in utility observed at twelve months over the remaining lifetime led to elevated ICERs that depending on choice of underlying EQ-5D scoring algorithm, remained cost-effective or exceeded the willingness-to-pay threshold.

In the two scenarios that assumed somewhat lower TAVI mortality (based on mortality observed in 12–24 month and 6–24-month periods of the trial) was maintained over the base case, incremental QALY gains increased markedly to 0.75 and 0.40 QALYs, while the incremental costs also increased, keeping the ICER reasonably stable at €3823 and €4323 per QALY gained. At the same time, the two hypothetical scenarios that explored the effect of potential higher long-term mortality of TAVI patients (RR = 1.03 and RR = 1.05 vs. general population mortality, while keeping SAVR at RR of 1.0) demonstrated that SAVR can be expected to become the dominant strategy if the long-term mortality risk in the TAVI strategy is around 4% higher than in the SAVR strategy (See Table 2 for detail on base case and scenarios).

**Table 2:** Cost-effectiveness results of base case and scenario analyses

	Effectiveness, QALY (LY)			Costs, €			ICER, €/QALY
	TAVI	SAVR	∆ TAVI-SAVR	TAVI	SAVR	∆ TAVI-SAVR	
Base case	9.34 (11.23)	9.21 (11.12)	0.13 (0.11)	52,267	51,433	833	6368
Base case (undiscounted)	11.39 (13.69)	11.24 (13.56)	0.15 (0.13)	59,913	58,872	1041	6895
15-year analysis horizon	8.24 (9.91)	8.12 (9.81)	0.12 (0.09)	46,361	45,653	708	5893
2-year analysis horizon (current trial follow-up)	1.63 (1.95)	1.58 (1.93)	0.06 (0.02)	30,156	30,029	127	2262
Consider all AEs in year 3 and following based on year 2 rates	9.34 (11.23)	9.21 (11.12)	0.13 (0.11)	58,025	57,136	890	6797
Utilities scored with EQ-5D-3L tariff	8.62 (11.23)	8.49 (11.12)	0.14 (0.11)	52,267	51,433	833	6133
Differential utility for SAVR and TAVI maintained over lifetime (EQ-5D-5L)	9.28 (11.23)	9.27 (11.12)	0.01 (0.11)	52,267	51,433	833	80,153
Differential utility for SAVR and TAVI maintained over lifetime (EQ-5D-3L)	8.60 (11.23)	8.51 (11.12)	0.08 (0.11)	52,267	51,433	833	9891
Lower long-term mortality for TAVI based on 12–24-month data (TAVI RR = 0.627, SAVR RR = 0.762) maintained over lifetime	10.77 (12.97)	10.02 (12.11)	0.75 (0.86)	56,739	53,877	2862	3823
Lower long-term mortality for TAVI based on 6–24-month data (TAVI RR = 0.711, SAVR RR = 0.773) maintained over lifetime	10.37 (12.49)	9.97 (12.06)	0.40 (0.44)	55,463	53,731	1732	4323
Higher mortality for TAVI in long-term projection (TAVI RR = 1.03; SAVR RR = 1.0 vs. lifetables)	9.25 (11.12)	9.21 (11.12)	0.04 (0.00)	51,986	51,433	553	15,177
Higher mortality for TAVI in long-term projection (TAVI RR 1.05; SAVR RR = 1.0 vs. lifetables)	9.18 (11.04)	9.21 (11.12)	−0.03 (−0.08)	51,806	51,433	372	SAVR dominant
Higher relative mortality of SAVR and TAVI in long-term projection (RR = 1.2 vs. lifetables)	8.76 (10.53)	8.64 (10.43)	0.13 (0.10)	50.588	49,807	781	6235
Lower relative mortality of SAVR and TAVI in long-term projection (RR = 0.751 vs. lifetables)	10.20 (12.28)	10.06 (12.17)	0.14 (0.l2)	54,922	54,009	912	6554

All costs and effects are discounted with 2.5% p.a

QALY, quality adjusted life year; LY, life year; ICER, incremental cost-effectiveness ratio; TAVI, transcatheter aortic valve implantation; SAVR, surgical aortic valve replacement

In deterministic one-way sensitivity analysis, the effect of variation in strategy-specific stroke rates had the largest impact on ICER, with TAVI ranging from dominance in cases where either the SAVR stroke rate was at the high range input or TAVI stroke rate at the low input, to ICERs between approx. €51,000 and €63,000 per QALY gained when SAVR stroke rates were low or TAVI stroke rates high, respectively. Variation in index costs led to scenarios ranging from TAVI dominance to an ICER of around €25,000 per QALY gained. Variation in all other parameters had a substantially more limited effect on the ICER, and in no scenario rendered TAVI not cost-effective (see supplementary materials). In threshold analysis, the TAVI strategy was found to remain cost-effective at the €50,000 per QALY gained threshold as long as its incremental cost vs. the SAVR strategy remained below €6540, that is, €5710 higher than the current incremental cost projection of €833 in the analysis base case.

**Fig. 3:**
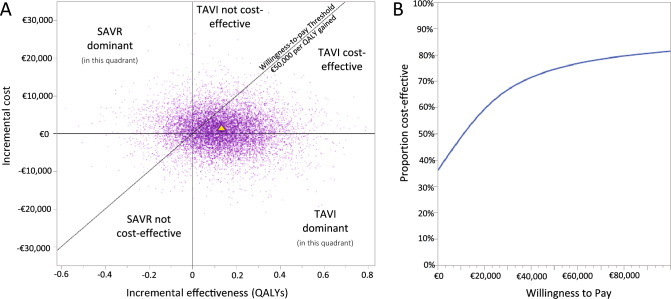
Results of Probabilistic Sensitivity Analysis. Scatter plot showing realizations of 10,000 Markov Chain Monte Carlo simulations and deterministic base case result (yellow triangle) (**A**), cost-effectiveness acceptability curve showing the likelihood of TAVI being cost-effective at different willingness-to-pay thresholds

Based on the 10,000 simulation runs of the PSA, TAVI was found to be dominant in 34.8% of simulations. When considering the €50,000 per QALY willingness-to-pay threshold as the upper bound for cost-effectiveness, TAVI was found dominant or cost-effective in 74.4% of simulation runs, with SAVR found dominant or cost-effective in the remaining 25.6% of simulations (Fig. 3).

### Model Validation

The review of model code and model projections did not identify inconsistencies. Changes in input parameters led to expected and plausible changes in projected cost and outcomes, confirming internal validity of the analysis model. Life expectancy for patients aged 74, per published data from the French government [17], is reported as 13.0 years for males and 15.8 years for females. In conjunction with NOTION data that confirm a low risk cohort can be expected to experience long-term survival similar to general population survival [18], this confirmed the external validity of survival projections in the analysis model, which in a predominantly male population projected 13.69 and 13.56 LYs for TAVI and SAVR, respectively. See supplementary materials for further detail.

## Discussion

This study presents the first cost-effectiveness analysis conducted in the European setting based on the Evolut Low Risk trial. Using the French healthcare system as the context of analysis, TAVI with the self-expanding Evolut R or Evolut Pro device was found to be associated with some limited gains in quality-adjusted survival over the lifetime analysis horizon, at a concurrent marginal increase in lifetime cost that rendered TAVI cost-effective. In probabilistic sensitivity analysis, TAVI was found cost-effective in close to 7500 of the 10,000 simulations run, underscoring the relative robustness of the cost-effectiveness. At the same time, these results document an expected finding: the relatively small improvement in nominal survival with TAVI—which did not reach statistical significance in the trial—can lead to a not insignificant proportion of simulations where SAVR is found to be associated with a higher QALY gain than TAVI. Overall, SAVR was found cost-effective or dominant in approximately 25% of simulations.

The lifetime projections of 9.34 and 9.21 (+ 0.13) discounted QALYs are in close keeping with projections in two earlier cost-effectiveness studies of self-expandable TAVI vs. SAVR in low-risk populations. Tam et al*.* studied the cost-effectiveness of TAVI in the Canadian context, and projected lifetime gains of 9.13 and 9.05 (+ 0.08) QALYs for TAVI and SAVR [19]. Based on Canadian costs, that study found TAVI to be likely cost-effective, albeit at a country-specific ICER that was less favorable than the ICER projected in the current study for the French context. Of note, Tam et al*.* did not have access to trial-collected EQ-5D data, so needed to rely on HRQoL assumptions from prior trials. This might explain the somewhat lower incremental QALY difference of 0.08 compared to 0.13 in the current study. Similarly, a cost-effectiveness study of the NOTION trial, based on 5-year clinical data, projected a lifetime incremental QALY gain of 0.09 (scenario ranges 0.07 to 0.19), in close keeping with the findings of the current analysis of Evolut Low Risk data. The NOTION cost-effectiveness study, in a Danish healthcare context, found TAVI cost effective, but not highly cost-effective, for their all comers, but predominantly low-risk population (STS-PROM score 3.0 ± 1.6%) [5].

Compared to prior cost-effectiveness studies in higher-risk cohorts, the projected QALY gains in the low-risk population are smaller. For example, cost-effectiveness studies based on the CoreValve High Risk trial projected gains of 0.32 and 0.41 QALYs over lifetime in the United States and Dutch context [4, 20]. This seems intuitive, as differences in periprocedural survival were much more pronounced in the high-risk cohort. Similarly, a cost-effectiveness study of balloon-expandable TAVI conducted for an intermediate risk population in the French setting based on PARTNER II data reported a lifetime gain of 0.41 QALYs [21]. Similarly to the findings in the current study, that study found TAVI and SAVR cost to be very similar, with even minimal savings for the TAVI strategy [21].

Among the strengths of the current study is its access to detailed patient-level data from the Evolut Low Risk trial, which facilitated a granular assessment of event proportions, health-related quality of life, and resource utilization, including data on strategy-specific utilization of rehabilitation. Also, detailed distributional information on all clinical event parameters could be derived from the trial data, enhancing the validity of the probabilistic simulations conducted in the PSA.

At the same time, the analysis is subject to several limitations. First, cost data—per French guidelines for analysis—were derived from national cost data collected in 2017, the latest available data year at the time of model development. As such, index procedure and hospitalization cost for the TAVI strategy are likely reflective of extreme and high-risk populations, as the intermediate and low-risk indications have only been approved more recently. Therefore, the cost estimate used in this analysis might over- rather than underestimate the cost of TAVI in low-risk patients. Also, evolving practice of TAVI in France might have led to further efficiency gains since 2017. For example, the reported national mean length of stay for transfemoral TAVI was more than one day shorter in 2020 compared to 2017 (7.34 vs. 8.64 days) [22, 23]. Further, ongoing procedural improvement such as the cusp overlap technique have been shown to reduce length of stay and need for pacemaker implantation [24–26]. All these factors might contribute to a further reduction in TAVI costs that could render TAVI cost saving, and thus the dominant treatment strategy. At the same time, stroke rates reported in the low-risk population are low. If there are neurologic deficits not formally adjudicated as stroke, some added cost might be incurred that are not captured. However, these would likely apply to both strategies and hence not change the incremental cost difference in any meaningful way. Further, any quality of life reduction associated with such additional deficit can be expected to already be captured in the study-derived utility data. Second, the current analysis does not take into consideration potential valve replacement costs at the end of device lifetimes. However, the NOTION trial recently reported continued valve function with no differences observed between the TAVI and SAVR cohorts at eight years, suggesting consideration of replacements would have negligible effect on incremental costs [18]. Nevertheless, any updated analyses should carefully consider lifetime management implications of both strategies based on then-available data with longer follow-up. This is particularly important in the low-risk population, and also includes future consideration of the role of valve-in-valve procedures. Third, no pacemaker replacements or pacemaker-associated quality of life differences were taken into consideration. However, contemporary pacemaker devices have lifetimes that commonly exceed the mean cohort survival projected in the current analysis and any quality-of-life implications of the higher pacemaker rate in the TAVI cohort can be expected to already be reflected in the trial-collected EQ-5D data. Fourth, while the trial-observed proportion of patients with disabling stroke was higher in the SAVR group than in the TAVI group, the analysis assumed the same unit costs of managing a stroke event, both acute and in long-term follow-up, thus taking a conservative approach that might under- rather than overestimate the TAVI cost-effectiveness. Fifth, the study data were derived from a multi-national randomized clinical trial and are specific to self-expandable TAVI devices. Real-world outcomes in France might differ somewhat from the trial-observed data. Nevertheless, the extensive sensitivity analyses conducted provide reasonable assurance about the robustness of the cost-effectiveness findings.

## Conclusion

TAVI using a self-expandable valve in patients at low surgical risk appears to be a cost-effective alternative to SAVR in the French healthcare system with an attractive health-economic value proposition. Longer follow-up data will help increase the accuracy of lifetime survival projections.

## Impact on daily practice

Emerging evidence suggests TAVI is a viable clinical alternative in aortic stenosis patients at low surgical risk with comparable outcomes to SAVR. The current study, conducted for a European setting based on two-year data from the Evolut Low Risk trial and French health system costs, suggests TAVI is a cost-effective treatment strategy that provides good value to healthcare payers. These study findings are in line with prior findings in high and intermediate risk populations.

### Supplementary Information

Below is the link to the electronic supplementary material.

Supplementary file1 (DOCX 123 KB)

## Data Availability

Not applicable.
