# Supplementary material for: Cost-effectiveness of transcatheter aortic valve implantation in patients at low surgical risk in France: a model-based analysis of the Evolut LR trial
Source: Eur J Health Econ. 2023 May 30;25(3):447–57. doi: 10.1007/s10198-023-01590-x (PMC10972970; doi:10.1007/s10198-023-01590-x)
Supplement: Supplementary file 1 — Supplementary file1 (DOCX 123 KB) [file 10198_2023_1590_MOESM1_ESM.docx]

**SUPPLEMENTARY MATERIALS**

**Cost-effectiveness of Transcatheter Aortic Valve Implantation in Patients at Low Surgical Risk in France: A Model-based Analysis of the Evolut LR Trial**

**S.1 Model Structure**

Shown below is a simplified graphical representation of the analysis model. The Markov structure is the same for both strategies. The primary health state stratification is survival without stroke, survival post stroke, and death. All other adverse events may occur in every cycle, and are accounted for cost-wise and, where applicable, disutility-wise. The health-related quality of life for the model states is derived directly from the trial data, with a strategy-specific EQ-5D value for the non-stroke states, and a combined utility estimate for the post-stroke state.


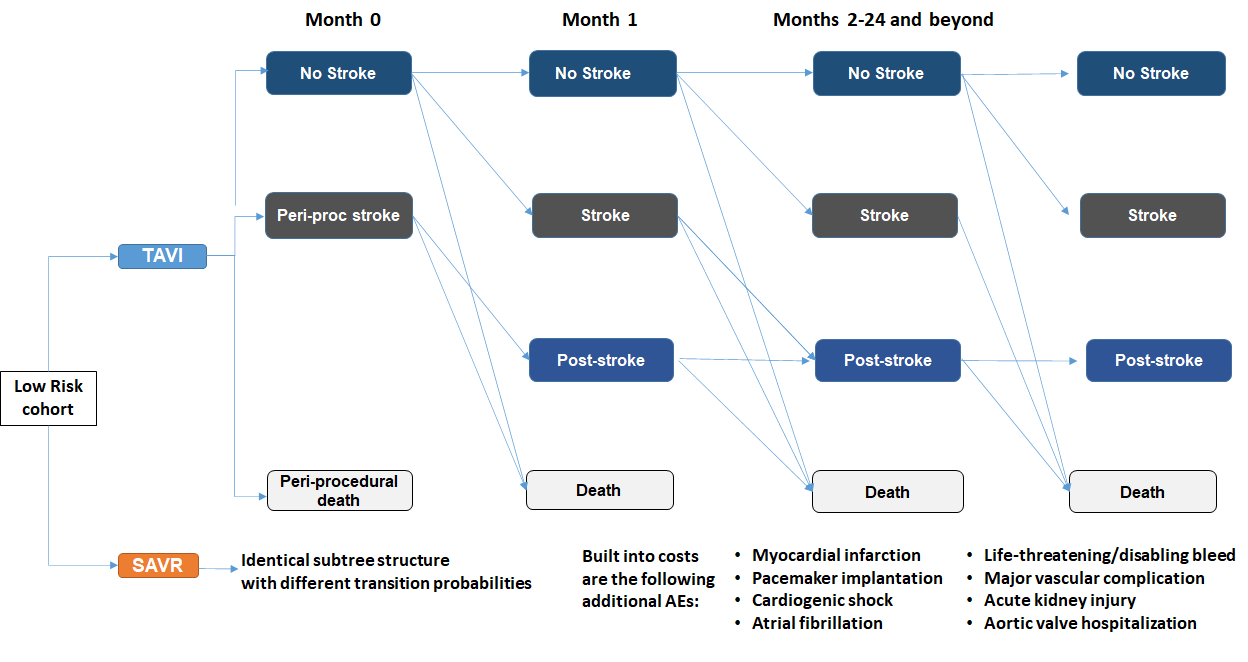


**Figure S.1.1.** Model schematic

**S.2 Further detail on parameter inputs**

**Clinical input parameters**

Clinical input parameters are shown in Table 1 of the manuscript. Note that parameters were informed by the most recent data set from the Evolut LR trial available at the time of analysis. While the Popma et al. 2019 publication provides 2-year data, the current analysis relies on additional follow-up data that have also formed the basis for the reimbursement submission to the French Government (see Haute Autorité de santé (HAS) assessment report “Avis Economique – Medtronic CoreValve Evolut R et Evolut Pro“, published May 2021^[[1]](#footnote-1)^). Differences between the Popma et al. and the more recent dataset are minimal.

**Cost input parameters:**

**Table S.2.1.** Cost inputs considered in the base case analysis: source used for methods and main assumptions

| Cost items | Unit costs | | Sources of information | Main assumptions & external validity |  |
| --- | --- | --- | --- | --- | --- |
|  | SAVR | TAVI |  |  |  |
| Index procedure | | | | | |
| Preoperative care | €3,111.60 | €3,140.86 | HAS Report on aortic valve (2011), HAS opinion on Sapien 3, (2020), experts’ opinion, Assurance Maladie, TNB, CCAM, Launois Report (2014) | Consideration of pulmonary function tests and dental and sinus scans as part of the preoperative examinations performed prior to TAVI surgery: conservative assumption, consistent with clinical guidelines and majority of experts opinion. |  |
| Procedure & index hospitalization | €23,323.97 | €23,743.05 | ENC-MCO 2017, ScanSanté, CEPS, Evolut LR study, LPPR | Cost estimations from ENC-MCO 2017 are considered representative of LR population. Cost estimations account for pacemaker implantation occurring during procedure & index hospitalization. |  |
| Rehabilitation costs | €652.27 | €74.24 | Evolut LR study, ENC-SSR 2018, experts’ opinion | Same type of rehabilitation stay is considered regardless of the procedure (SAVR or TAVI): conservative assumption. However, proportion of rehabilitation stays is specific for either SAVR or TAVI: consistent with experts’ opinion and trial data. |  |
| Outpatient follow-up | | | | | |
| Month 1 | €237.01 | | French payer tariffs (*Assurance Maladie*), TNB, CCAM, BdM_IT | Post-operative follow-up is considered the same for SAVR and TAVI, based on experts’ opinion. |  |
| Month 6 | €60.00 | |  |  |  |
| Month 12 | €18.42 | |  |  |  |
| Annually beyond 12 months | €261.85 | |  |  |  |
| Adverse events | | | | | |
| Stroke | €5,857.48 | | Evolut LR study, ENC-MCO 2017 | Cost of AE (excluding stroke and mortality) is considered only in the first two years in the analysis.  Except from aortic valve rehospitalization and part of pacemaker implantation, costs of AE observed at 30-day timepoint in the trial are considered to be covered within procedure & index hospitalization cost. |  |
| Myocardial infarction | €5,776.16 | |  |  |  |
| Permanent pacemaker implantation | €6,734.30 | |  |  |  |
| Bleeding event | €11,282.45 | |  |  |  |
| Cardiogenic shock | €4,788.74 | |  |  |  |
| Acute kidney injury | €4,588.56 | |  |  |  |
| Major vascular complication | €7,511.48 | |  |  |  |
| Atrial fibrillation | €3,607.76 | |  |  |  |
| Aortic valve rehospitalization | €4,788.74 | |  |  |  |
| Reintervention (surgical) | €23,323.97 | |  |  |  |
| Adverse event follow-up | | | | | |
| Stroke (monthly) | €710.82 | | De Pouvourville (2016) | Only AE which have extensive impact in terms of long-term costs and utilities were considered in the AE follow-up cost item (stroke and pacemaker implantation). |  |
| Pacemaker implantation – year 1 (annually) | €545.16 | | HAS, experts’ opinion |  |  |
| Pacemaker implantation – year 2+ (annually) | €272.58 | |  |  |  |
| Other cost items | | | | | |
| Transportation | €73.43 | | Cour des comptes (2011, 2019), LegiFrance (26/02/2020), Commission des comptes de la sécurité sociale (07/2016) | Only transportation to hospital in case of AE and index procedure is accounted for in the analysis |  |
| End-of-life | €7,202.04 | | Poulalhon et al. (2017), ENC-MCO 2017, ENC-SSR 2018, ENC-HAD 2018 | The analysis considers deaths in MCO, SSR and HAD |  |

AE: adverse event; BdM_IT: French database reporting medicines public price (*Base des médicaments et informations tarifaires*); CCAM: tariffs and classification of medical acts (*Classification commune des actes médicaux*); CEPS: French payer (*Comité Économique des Produits de Santé*); DRG: diagnostic related group, ENC: French national cost study (*Etude Nationale des Coûts*) which provides cost data per diagnosis-related group (DRG) from a representative sample of public and private hospitals; HAD: home-based hospitalization (*Hospitalisation à domicile*); HAS: French health technology assessment body (*Haute Autorité de Santé*), LPPR: French database reporting medical devices public price (*Liste des produits et prestations remboursables*); LR: low risk, MCO: medicine-surgery-obstetrics *(médecine-chirurgie-obstétrique);* SAVR: surgical aortic valve replacement; SSR: rehabilitation (*soins de suites et de réadaptation*); TAVI: transcatheter aortic valve implantation; TNB: French database reporting biological act tariffs (*Table Nationale de Biologie*).

Note: Pacemaker implantations observed at 30 days and having occurred within the hospitalization stay in Evolut LR trial were valued through the TAVI implantation DRG (as per French tariffication rules), considering the additional pacemaker acquisition cost. Pacemaker implantations occurring outside the hospitalization stay in Evolut LR were valued separately, through a dedicated DRG.

**Table S.2.2.** Full listing of all model parameters, including base case value, lower and upper bound, and distributional information for probabilistic sensitivity analysis

| Parameter Name | Basecase value | Lower Bound | Upper Bound | Distribution | Parameter A | Parameter B |
| --- | --- | --- | --- | --- | --- | --- |
| Pre-op costs TAVI | € 3,140.86 | € 2,895.71 | € 3,252.90 | Gamma | 309.29 | 10.16 |
| Pre-op costs SAVR | € 3,111.60 | € 2,933.65 | € 3,290.84 | Gamma | 303.55 | 10.25 |
| Administration costs TAVI | € 23,743.05 | € 21,368.75 | € 26,117.36 | Gamma | 100.00 | 237.43 |
| Administration costs SAVR | € 23,323.97 | € 20,991.58 | € 25,656.37 | Gamma | 100.00 | 233.24 |
| Rehab cost TAVI | € 74.24 | € 71.23 | € 77.26 | Gamma | 606.11 | 0.12 |
| Rehab cost SAVR | € 652.27 | € 625.77 | € 678.76 | Gamma | 606.11 | 1.08 |
| Folllow-up cost TAVI & SAVR, 30d | € 237.01 | € 213.31 | € 260.71 | Gamma | 100.00 | 2.37 |
| Folllow-up cost TAVI & SAVR, 6M | € 60.00 | € 54.00 | € 66.00 | Gamma | 100.00 | 0.60 |
| Folllow-up cost TAVI & SAVR, 1 yr. | € 18.42 | € 16.58 | € 20.26 | Gamma | 100.00 | 0.18 |
| Folllow-up cost TAVI & SAVR, annually yr. 2+ | € 261.85 | € 235.67 | € 288.04 | Gamma | 100.00 | 2.62 |
| Monthly stroke follow-up costs, TAVI & SAVR | € 710.82 | € 497.57 | € 924.18 | Gamma | 11.10 | 64.01 |
| Pacemaker follow-up costs, first year | € 545.16 | € 490.64 | € 599.68 | Gamma | 100.00 | 5.45 |
| Pacemaker follow-up costs, year 2+ | € 272.58 | € 245.32 | € 299.84 | Gamma | 100.00 | 2.73 |
| Cost of stroke AE (beyond index) | € 5,857.48 | € 5,514.48 | € 6,202.57 | Gamma | 289.86 | 20.21 |
| Cost of MI AE (beyond index) | € 5,776.16 | € 5,395.85 | € 6,156.96 | Gamma | 230.37 | 25.07 |
| Cost of permanent pacemaker implantation (beyond index) | € 6,734.30 | € 6,060.87 | € 7,407.73 | Gamma | 100.00 | 67.34 |
| Cost of bleeding event (beyond index) | € 11,282.45 | € 10,152.74 | € 12,412.16 | Gamma | 99.74 | 113.12 |
| Cost of cardiogenic shock (beyond index) | € 4,788.74 | € 4,550.90 | € 5,026.59 | Gamma | 405.37 | 11.81 |
| Cost of AKI (beyond index) | € 4,588.56 | € 4,156.00 | € 5,025.53 | Gamma | 111.39 | 41.19 |
| Cost of major vascular complication (beyond index) | € 7,511.48 | € 6,932.37 | € 8,091.93 | Gamma | 167.85 | 44.75 |
| Cost of atrial fibrillation (beyond index) | € 3,607.76 | € 3,109.91 | € 4,113.29 | Gamma | 51.71 | 69.76 |
| Cost of aortic valve rehospitalization (beyond index) | € 4,788.74 | € 4,550.90 | € 5,026.59 | Gamma | 405.37 | 11.81 |
| Cost of reintervention (surgical) (beyond index) | € 23,323.97 | € 20,991.58 | € 25,656.37 | Gamma | 100.00 | 233.24 |
| End-of-life costs | € 7,202.04 | € 6,725.14 | € 7,678.93 | Gamma | 228.07 | 31.58 |
| Cost of transportation | € 73.43 | € 66.09 | € 80.78 | Gamma | 100.00 | 0.73 |
| Cohort age | 73.9 | 66 | 81 | Normal | 100.00 | 0.74 |
| Gender (% female) | 0.353 | 0.0000 | 1.0000 | Beta | 805.87 | 1,477.04 |
| Stroke 30d - SAVR | 0.032 | 0.0191 | 0.0483 | Beta | 17.59 | 532.09 |
| Stroke 30d - Corevalve Evolut R/PRO | 0.034 | 0.0212 | 0.0500 | Beta | 19.82 | 563.07 |
| Myocardial infarction 30d - SAVR | 0.0132 | 0.0050 | 0.0245 | Beta | 6.86 | 513.17 |
| Myocardial infarction 30d - Corevalve Evolut R/PRO | 0.0082 | 0.0022 | 0.0172 | Beta | 4.16 | 503.14 |
| Permanent pacemaker implantation 30d - SAVR | 0.0599 | 0.0418 | 0.0810 | Beta | 33.67 | 528.45 |
| Permanent pacemaker implantation 30d - Corevalve Evolut R/PRO | 0.171 | 0.1436 | 0.1991 | Beta | 123.51 | 598.75 |
| Proportion of PPI during first month in separate admission - SAVR | 0.0488 | 0.0316 | 0.0705 | Beta | 22.59 | 440.42 |
| Proportion of PPI during first month in separate admission - Corevalve EvolutR/PRO | 0.0960 | 0.0772 | 0.1166 | Beta | 83.22 | 783.62 |
| Bleeding event (major or life-threatening/disabling) 30d - SAVR | 0.1038 | 0.0807 | 0.1299 | Beta | 61.69 | 532.67 |
| Bleeding event (major or life-threatening/disabling) 30d - Corevalve Evolut R/PRO | 0.0658 | 0.0493 | 0.0847 | Beta | 40.38 | 573.32 |
| Cardiogenic shock 30d - SAVR | 0.0000 | 0.0000 | 0.0050 | N/A | N/A | N/A |
| Cardiogenic shock 30d - Corevalve Evolut R/PRO | 0.0000 | 0.0000 | 0.0050 | N/A | N/A | N/A |
| AKI 30d - SAVR | 0.1009 | 0.0796 | 0.1246 | Beta | 91.43 | 814.76 |
| AKI 30d - Corevalve Evolut R/PRO | 0.0205 | 0.0109 | 0.0342 | Beta | 11.41 | 545.36 |
| Major vascular complication 30d - SAVR | 0.0307 | 0.0188 | 0.0461 | Beta | 18.61 | 587.68 |
| Major vascular complication 30d - Corevalve Evolut R/PRO | 0.0370 | 0.0246 | 0.0520 | Beta | 26.87 | 699.30 |
| Atrial fibrillation 30d - SAVR | 0.3538 | 0.3200 | 0.3886 | Beta | 263.77 | 481.76 |
| Atrial fibrillation 30d - Corevalve Evolut R/PRO | 0.0753 | 0.0566 | 0.0960 | Beta | 52.36 | 642.94 |
| Reintervention 30d - SAVR | 0.0030 | 0.0000 | 0.0125 | Beta | 0.73 | 242.43 |
| Reintervention 30d - Corevalve Evolut R/PRO | 0.0030 | 0.0017 | 0.0046 | Beta | 0.99 | 330.34 |
| Myocardial infarction, month 2-12 - SAVR | 0.0028 | 0.0019 | 0.0039 | Beta | 32.22 | 11,302.90 |
| Myocardial infarction, month 2-12 - Corevalve Evolut R/PRO | 0.0098 | 0.0081 | 0.0120 | Beta | 94.72 | 9,589.44 |
| Permanent pacemaker implantation, month 2-12 - SAVR | 0.0081 | 0.0061 | 0.0101 | Beta | 64.41 | 7,928.13 |
| Permanent pacemaker implantation, month 2-12 - Corevalve Evolut R/PRO | 0.0188 | 0.0153 | 0.0231 | Beta | 86.38 | 4,516.35 |
| Bleeding event (major or life-threatening/disabling), month 2-12 - SAVR | 0.0182 | 0.0143 | 0.0222 | Beta | 81.27 | 4,384.63 |
| Bleeding event (major or life-threatening/disabling), month 2-12 - Corevalve Evolut R/PRO | 0.0152 | 0.0113 | 0.0192 | Beta | 57.21 | 3,695.32 |
| Cardiogenic shock, month 2-12 - SAVR | 0.0000 | 0.0000 | 0.0050 | N/A | N/A | N/A |
| Cardiogenic shock, month 2-12 - Corevalve Evolut R/PRO | 0.0000 | 0.0000 | 0.0050 | N/A | N/A | N/A |
| AKI, month 2-12 - SAVR | 0.0001 | 0.0000 | 0.0050 | N/A | N/A | N/A |
| AKI, month 2-12 - Corevalve Evolut R/PRO | 0.0005 | 0.0000 | 0.0050 | Beta | 0.00 | 3.52 |
| Major vascular complication, month 2-12 - SAVR | 0.0033 | 0.0024 | 0.0044 | Beta | 43.37 | 13,105.10 |
| Major vascular complication, month 2-12 - Corevalve Evolut R/PRO | 0.0000 | 0.0000 | 0.0050 | N/A | N/A | N/A |
| Atrial fibrillation, month 2-12 - SAVR | 0.0332 | 0.0322 | 0.0342 | Beta | 4,262.25 | 124,123.42 |
| Atrial fibrillation, month 2-12 - Corevalve Evolut R/PRO | 0.0187 | 0.0168 | 0.0206 | Beta | 341.59 | 17,966.84 |
| Aortic valve rehospitalization, year 1 - SAVR | 0.0590 | 0.0409 | 0.0803 | Beta | 32.70 | 521.49 |
| Aortic valve rehospitalization, year 1 - Corevalve Evolut R/PRO | 0.0330 | 0.0199 | 0.0492 | Beta | 18.69 | 547.62 |
| Reintervention, month 2-12 - SAVR | 0.0010 | 0.0003 | 0.0022 | Beta | 4.00 | 3,991.01 |
| Reintervention, month 2-12 - Corevalve Evolut R/PRO | 0.0030 | 0.0017 | 0.0046 | Beta | 15.95 | 5,300.38 |
| Myocardial infarction, month 13-24 - SAVR | 0.0000 | 0.0000 | 0.0050 | N/A | N/A | N/A |
| Myocardial infarction, month 13-24 - Corevalve Evolut R/PRO | 0.0030 | 0.0001 | 0.0110 | Beta | 0.99 | 330.34 |
| Permanent pacemaker implantation, month 13-24 - SAVR | 0.0090 | 0.0020 | 0.0213 | Beta | 3.20 | 352.56 |
| Permanent pacemaker implantation, month 13-24 - Corevalve Evolut R/PRO | 0.0280 | 0.0152 | 0.0449 | Beta | 13.52 | 469.32 |
| Bleeding event (major or life-threatening/disabling), month 13-24 - SAVR | 0.0150 | 0.0068 | 0.0266 | Beta | 8.85 | 581.15 |
| Bleeding event (major or life-threatening/disabling), month 13-24 - Corevalve Evolut R/PRO | 0.0230 | 0.0142 | 0.0338 | Beta | 20.65 | 877.19 |
| Major vascular complication, month 13-24 - SAVR | 0.0020 | 0.0000 | 0.0091 | Beta | 0.64 | 317.72 |
| Major vascular complication, month 13-24 - Corevalve Evolut R/PRO | 0.0020 | 0.0000 | 0.0091 | Beta | 0.64 | 317.72 |
| Atrial fibrillation, month 13-24 - SAVR | 0.0100 | 0.0012 | 0.0270 | Beta | 2.01 | 199.03 |
| Atrial fibrillation, month 13-24 - Corevalve Evolut R/PRO | 0.0280 | 0.0190 | 0.0380 | Beta | 30.45 | 1,057.19 |
| Aortic valve rehospitalization, month 13-24 - SAVR | 0.0130 | 0.0050 | 0.0240 | Beta | 6.66 | 505.58 |
| Aortic valve rehospitalization, month 13-24 - Corevalve Evolut R/PRO | 0.0160 | 0.0090 | 0.0250 | Beta | 15.73 | 967.27 |
| Reintervention, month 13-24 - SAVR | 0.0050 | 0.0009 | 0.0124 | Beta | 2.76 | 549.02 |
| Reintervention, month 13-24 - Corevalve Evolut R/PRO | 0.0030 | 0.0002 | 0.0094 | Beta | 1.43 | 476.13 |
| Disutility permanent pacemaker implant | 0.0030 | 0.0000 | 0.0310 | Beta | 0.09 | 28.82 |
| Disutility MI | 0.0409 | 0.0240 | 0.0630 | Beta | 16.00 | 375.27 |
| Disutility Stroke | 0.1610 | 0.0760 | 0.2710 | Beta | 8.54 | 44.49 |
| Multiplier to vary trial-observed utilities - SAVR | 1.0 | 0.9750 | 1.0250 | Normal |  |  |
| Multiplier to vary trial-observed utilities - Corevalve Evolut R/PRO | 1.0 | 0.9750 | 1.0250 | Normal |  |  |
| Multiplier to vary trial-observed stroke rates - SAVR | 1.0 | 0.6500 | 1.5167 | Gamma | 25 | 0.04 |
| Multiplier to vary trial-observed stroke rates - CoreValve Evolut R/PRO | 1.0 | 0.6935 | 1.4516 | Gamma | 25 | 0.04 |
| Multiplier to vary trial-observed mortality rates - SAVR | 1.0 | 0.6136 | 1.6364 | Normal |  |  |
| Multiplier to vary trial-observed mortality rates - Corevalve Evolut R/PRO | 1.0 | 0.6000 | 1.6571 | Normal |  |  |
| Discount rate costs (% p.a.) | 0.0250 | 0.0000 | 0.1000 | Gamma | 100 | 0.00025 |
| Discount rate effects (% p.a.) | 0.0250 | 0.0000 | 0.1000 | Gamma | 100 | 0.00025 |

AKI: acute kidney injury; MI: myocardial infarction; PPI: permanent pacemaker implantation; SAVR: surgical aortic valve replacement; TAVI: transcatheter aortic valve implantation.

**S.3 Additional Detail on Long-term Mortality Projections**

As outlined in the manuscript, mortality was modeled “along-the-trial” through two years follow-up. In the base case, long-term survival was modeled assuming both cohorts had survival equivalent to general population (relative risk (RR) of 1.0 vs. French lifetable data). As the RR for SAVR and TAVI versus lifetables was 0.751 in period 6-18 months, this RR of 0.751 was assumed for month 25 and then subsequently faded out to a RR of 1.0 at 36 months, after which the RR of 1.0 was maintained over lifetime.

Other scenarios explored in sensitivity analyses assumed strategy-specific RRs vs. lifetables that were maintained over the lifetime. These included the following scenarios:

1. Lower long-term mortality for TAVI based on 12–24-month mortality data (TAVI RR=0.627, SAVR RR=0.762). These strategy-specific RRs versus lifetables were then maintained over lifetime.
2. Lower long-term mortality for TAVI based on 6–24-month data (TAVI RR=0.711, SAVR RR=0.773). These strategy-specific RRs versus lifetables were then maintained over lifetime.
3. Higher mortality for TAVI in long-term projection (TAVI RR=1.03; SAVR RR=1.0 vs. lifetables), maintained over the remaining lifetime. These strategy-specific RRs versus lifetables were then maintained over lifetime. This scenario was the first of two hypothetical scenarios explored. As opposed to the earlier scenarios involving TAVI, no trial-derived RRs could be derived through 24-month follow-up that would have supported this scenario.
4. Higher mortality for TAVI in long-term projection (TAVI RR 1.05; SAVR RR=1.0 vs. lifetables), maintained over the remaining lifetime. Similar to the prior scenario, this was a hypothetical scenario explored.
5. Higher relative mortality of SAVR and TAVI in long-term projection (RR=1.2 vs. lifetables). This scenario is again hypothetical, but in line with some assumptions made in earlier cost-effectiveness studies about long-term mortality (albeit for different surgical risk strata). The analysis was primarily performed for exploratory purposes.
6. Lower relative mortality of SAVR and TAVI in long-term projection (RR=0.751 vs. lifetables), maintained over lifetime. Again, this analysis was performed for exploratory purposes only.

See main manuscript Table 2 for respective analysis results.

**S.4 Deterministic sensitivity analysis results**

Shown below are the results of deterministic sensitivity analysis. Figure S.4.1 shows a Tornado diagram with the ten most influential parameters. Table S.4.1 shows the results for all tested parameters.


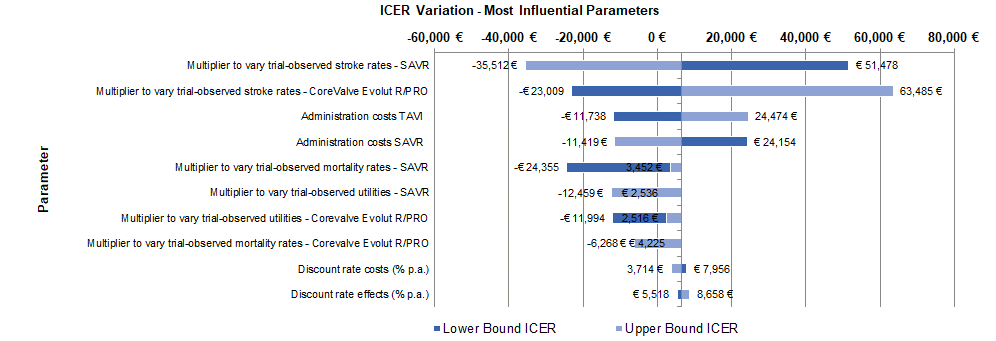


**Figure S.4.1.** Tornado diagram depicting the ten most influential parameters

**Table S.3.1.** Full listing of deterministic sensitivity analysis results

| **Parameter Name** | **Lower Bound ICER** | **Upper Bound ICER** | **ICER Difference** |
| --- | --- | --- | --- |
| Multiplier to vary trial-observed stroke rates - SAVR | € 51,477.92 | (€ 35,512.43) | € 86,990.35 |
| Multiplier to vary trial-observed stroke rates - CoreValve Evolut R/PRO | (€ 23,008.86) | € 63,484.77 | € 86,493.64 |
| Administration costs TAVI | (€ 11,738.18) | € 24,473.95 | € 36,212.13 |
| Administration costs SAVR | € 24,154.47 | (€ 11,418.70) | € 35,573.18 |
| Multiplier to vary trial-observed mortality rates - SAVR | (€ 24,355.40) | € 3,452.03 | € 27,807.43 |
| Multiplier to vary trial-observed utilities - SAVR | € 2,535.91 | (€ 12,459.43) | € 14,995.34 |
| Multiplier to vary trial-observed utilities - Corevalve Evolut R/PRO | (€ 11,994.34) | € 2,516.05 | € 14,510.39 |
| Multiplier to vary trial-observed mortality rates - Corevalve Evolut R/PRO | € 4,224.86 | (€ 6,267.60) | € 10,492.46 |
| Discount rate costs (% p.a.) | € 7,955.90 | € 3,714.14 | € 4,241.77 |
| Discount rate effects (% p.a.) | € 5,518.43 | € 8,658.34 | € 3,139.90 |
| Pre-op costs SAVR | € 7,727.71 | € 4,998.23 | € 2,729.48 |
| Pre-op costs TAVI | € 4,494.53 | € 7,224.01 | € 2,729.48 |
| Permanent pacemaker implantation 30d - Corevalve Evolut R/PRO | € 5,021.60 | € 7,747.07 | € 2,725.47 |
| Permanent pacemaker implantation, month 13-24 - Corevalve Evolut R/PRO | € 5,430.06 | € 7,615.83 | € 2,185.77 |
| Reintervention, month 13-24 - SAVR | € 7,048.80 | € 5,131.80 | € 1,917.00 |
| Permanent pacemaker implantation 30d - SAVR | € 7,228.02 | € 5,365.30 | € 1,862.72 |
| Bleeding event (major or life-threatening/disabling), month 13-24 - Corevalve Evolut R/PRO | € 5,635.91 | € 7,270.19 | € 1,634.28 |
| Bleeding event (major or life-threatening/disabling), month 13-24 - SAVR | € 7,034.16 | € 5,419.12 | € 1,615.03 |
| Reintervention, month 13-24 - Corevalve Evolut R/PRO | € 5,898.85 | € 7,444.94 | € 1,546.10 |
| Aortic valve rehospitalization, year 1 - SAVR | € 7,052.39 | € 5,546.40 | € 1,506.00 |
| Permanent pacemaker implantation, month 13-24 - SAVR | € 6,869.80 | € 5,477.81 | € 1,391.99 |
| Monthly stroke follow-up costs, TAVI & SAVR | € 5,703.94 | € 7,032.19 | € 1,328.26 |
| Cohort age | € 6,948.91 | € 5,721.58 | € 1,227.33 |
| Aortic valve rehospitalization, year 1 - Corevalve Evolut R/PRO | € 5,883.55 | € 6,977.24 | € 1,093.69 |
| Bleeding event (major or life-threatening/disabling), month 2-12 - Corevalve Evolut R/PRO | € 6,030.43 | € 6,706.16 | € 675.73 |
| Bleeding event (major or life-threatening/disabling), month 2-12 - SAVR | € 6,699.70 | € 6,026.63 | € 673.07 |
| Aortic valve rehospitalization, month 13-24 - SAVR | € 6,645.67 | € 5,982.24 | € 663.43 |
| Permanent pacemaker implantation, month 2-12 - Corevalve Evolut R/PRO | € 6,113.70 | € 6,682.83 | € 569.13 |
| Aortic valve rehospitalization, month 13-24 - Corevalve Evolut R/PRO | € 6,121.52 | € 6,687.23 | € 565.71 |
| Pacemaker follow-up costs, year 2+ | € 6,090.17 | € 6,645.60 | € 555.43 |
| Reintervention, month 2-12 - Corevalve Evolut R/PRO | € 6,145.72 | € 6,647.69 | € 501.97 |
| Major vascular complication, month 13-24 - Corevalve Evolut R/PRO | € 6,259.83 | € 6,753.34 | € 493.51 |
| Major vascular complication, month 13-24 - SAVR | € 6,474.93 | € 5,987.35 | € 487.59 |
| Myocardial infarction, month 13-24 - Corevalve Evolut R/PRO | € 6,245.60 | € 6,705.39 | € 459.78 |
| Cost of permanent pacemaker implantation (beyond index) | € 6,150.71 | € 6,585.07 | € 434.36 |
| Rehab cost SAVR | € 6,567.50 | € 6,168.27 | € 399.23 |
| Folllow-up cost TAVI & SAVR, annually yr. 2+ | € 6,184.09 | € 6,551.69 | € 367.60 |
| Folllow-up cost TAVI & SAVR, 30d | € 6,188.26 | € 6,547.52 | € 359.26 |
| Reintervention, month 2-12 - SAVR | € 6,491.96 | € 6,164.41 | € 327.55 |
| Gender (% female) | € 6,270.78 | € 6,566.08 | € 295.31 |
| Permanent pacemaker implantation, month 2-12 - SAVR | € 6,505.76 | € 6,222.81 | € 282.95 |
| Major vascular complication, month 2-12 - Corevalve Evolut R/PRO | € 6,367.12 | € 6,649.14 | € 282.03 |
| Myocardial infarction, month 13-24 - SAVR | € 6,367.89 | € 6,160.38 | € 207.51 |
| Proportion of PPI during first month in separate admission - Corevalve EvolutR/PRO | € 6,275.06 | € 6,469.60 | € 194.54 |
| Cardiogenic shock, month 2-12 - Corevalve Evolut R/PRO | € 6,367.89 | € 6,548.68 | € 180.79 |
| Cardiogenic shock, month 2-12 - SAVR | € 6,367.89 | € 6,188.20 | € 179.69 |
| Cardiogenic shock, month 13-24 - Corevalve Evolut R/PRO | € 6,367.89 | € 6,542.10 | € 174.21 |
| AKI, month 2-12 - Corevalve Evolut R/PRO | € 6,352.25 | € 6,525.60 | € 173.35 |
| Cardiogenic shock, month 13-24 - SAVR | € 6,367.89 | € 6,195.41 | € 172.48 |
| AKI, month 2-12 - SAVR | € 6,372.11 | € 6,199.82 | € 172.29 |
| Myocardial infarction, month 2-12 - Corevalve Evolut R/PRO | € 6,296.16 | € 6,466.51 | € 170.35 |
| AKI, month 13-24 - Corevalve Evolut R/PRO | € 6,367.89 | € 6,534.93 | € 167.04 |
| AKI, month 13-24 - SAVR | € 6,367.89 | € 6,202.51 | € 165.38 |
| Major vascular complication, month 2-12 - SAVR | € 6,419.44 | € 6,308.29 | € 111.14 |
| Cost of atrial fibrillation (beyond index) | € 6,421.90 | € 6,313.04 | € 108.87 |
| Atrial fibrillation, month 2-12 - Corevalve Evolut R/PRO | € 6,314.86 | € 6,422.34 | € 107.48 |
| Pacemaker follow-up costs, first year | € 6,314.83 | € 6,420.94 | € 106.11 |
| Folllow-up cost TAVI & SAVR, 6M | € 6,323.19 | € 6,412.58 | € 89.39 |
| Cost of bleeding event (beyond index) | € 6,325.04 | € 6,410.73 | € 85.69 |
| Myocardial infarction, month 2-12 - SAVR | € 6,406.96 | € 6,321.44 | € 85.51 |
| Cost of aortic valve rehospitalization (beyond index) | € 6,409.92 | € 6,325.85 | € 84.07 |
| Proportion of PPI during first month in separate admission - SAVR | € 6,397.31 | € 6,330.61 | € 66.69 |
| Cost of MI AE (beyond index) | € 6,339.97 | € 6,395.84 | € 55.87 |
| Atrial fibrillation, month 2-12 - SAVR | € 6,394.93 | € 6,340.67 | € 54.26 |
| Rehab cost TAVI | € 6,344.98 | € 6,390.79 | € 45.80 |
| Cost of major vascular complication (beyond index) | € 6,381.85 | € 6,353.89 | € 27.96 |
| Follow-up cost TAVI & SAVR, 1 yr. | € 6,354.44 | € 6,381.33 | € 26.89 |
| End-of-life costs | € 6,377.51 | € 6,358.27 | € 19.24 |
| Cost of stroke AE (beyond index) | € 6,364.32 | € 6,371.48 | € 7.16 |
| Atrial fibrillation, month 13-24 - SAVR | € 6,365.57 | € 6,372.41 | € 6.84 |
| Cost of reintervention (surgical) (beyond index) | € 6,365.76 | € 6,370.02 | € 4.26 |
| Cost of AKI (beyond index) | € 6,366.83 | € 6,368.96 | € 2.13 |
| Cost of transportation | € 6,366.87 | € 6,368.90 | € 2.03 |

AKI: acute kidney injury; MI: myocardial infarction; PPI: permanent pacemaker implantation; SAVR: surgical aortic valve replacement; TAVI: transcatheter aortic valve implantation.

1. <https://www.has-sante.fr/upload/docs/application/pdf/2021-07/corevalve_evolut_r_evolut_pro_11052021_avis_economique.pdf> [↑](#footnote-ref-1)
